# Surgical Management of Cutaneous Neoplasms: Biopsy Strategies, Margin Assessment, and Special Considerations

**DOI:** 10.3390/dermatopathology13030034

**Published:** 2026-07-23

**Authors:** Maged Daruish, Catherine M. Stefanato

**Affiliations:** Department of Dermatopathology, St. John’s Institute of Dermatology, Guy’s and St. Thomas’ NHS Trust, London SE1 7EH, UK; maged.daruish@nhs.net

**Keywords:** biopsy techniques, cutaneous tumours, malignant melanoma

## Abstract

There has been a rising incidence of both melanoma and non-melanoma skin cancers, requiring knowledge of effective management techniques. This review aims to provide a structured approach to biopsy selection, surgical excision, and margin assessment for common cutaneous neoplasms.

## 1. Introduction

The increasing burden of cutaneous neoplasms necessitates knowledge of effective management strategies. There is an increasing incidence rate of both melanoma and non-melanoma skin cancers (NMSCs) in the UK and Europe. Europe has the highest number of melanoma cases worldwide and the second highest number of NMSCs [1]. Melanoma is the fifth most common cancer in the UK, with approximately 18,321 melanoma cases diagnosed annually and an increasing incidence by 154% since the early 1990s [2]. Similarly, NMSCs have risen 169%, with about 156,000 new cases per year [3].

Management of skin cancers is a stepwise process from clinical suspicion to definitive treatment. Clear, effective management hinges on appropriate initial biopsy, understanding margin guidelines, and adapting techniques for specific tumour types.

This review is intended for both clinicians and pathologists. We discuss the pros and cons of the various skin sampling techniques, as well as margin guidelines for common skin neoplasms. Our review aims to bridge the gap between clinician procedural guidance and pathology-informed margin discussion.

## 2. Biopsy Techniques

**Figure 1:**
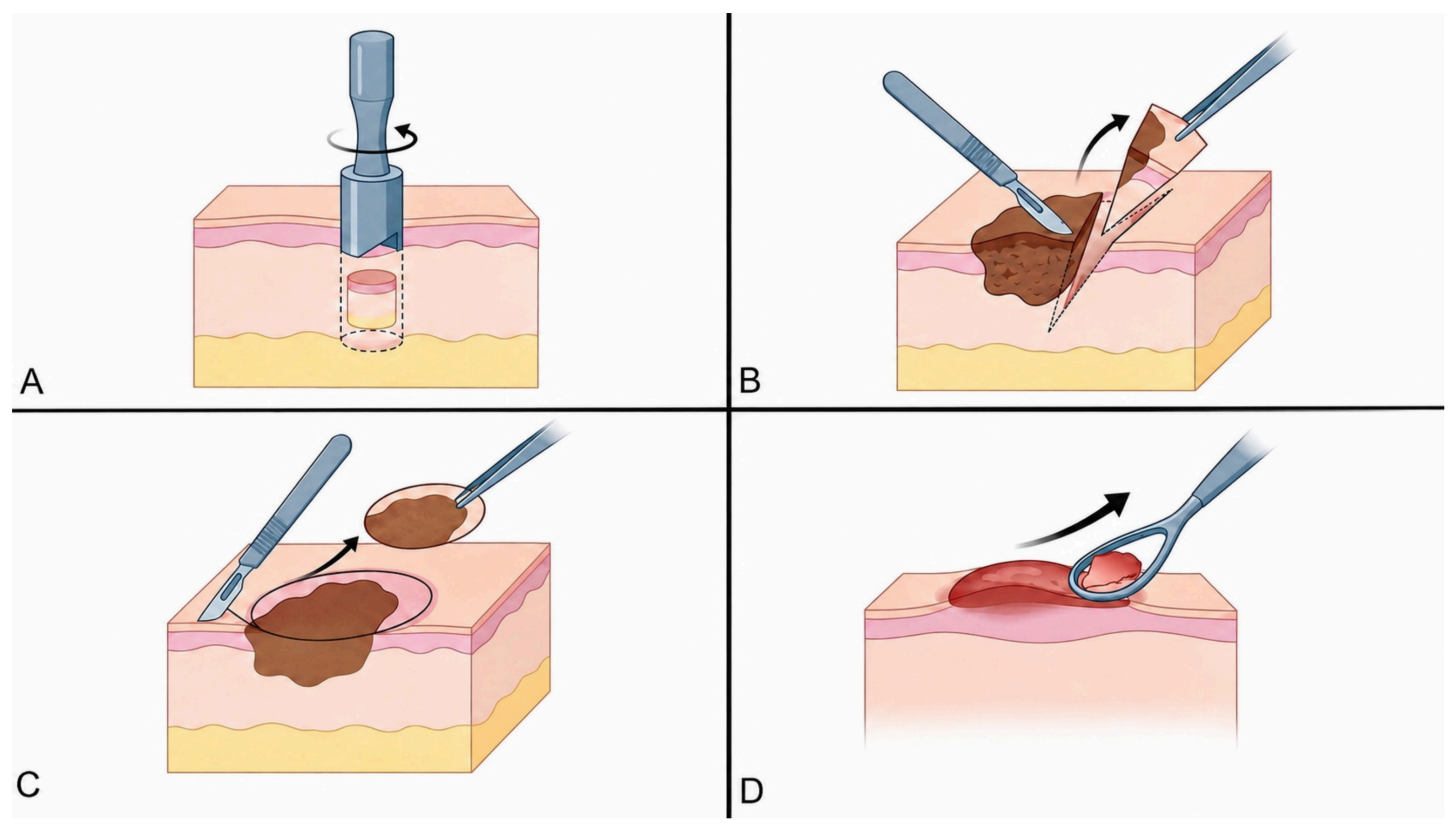
Common skin biopsy techniques: (**A**) punch biopsy: circular punch tool removes a cylindrical core of tissue extending through the epidermis and dermis. (**B**) Incisional biopsy: sampling a representative portion of the lesion for histopathological assessment. (**C**) Excisional biopsy: the entire lesion is removed with a surrounding margin of clinically normal tissue. (**D**) Curettage: superficial removal of raised lesional tissue using a curette. (Created with FIGURELABS.AI).

**Figure 2:**
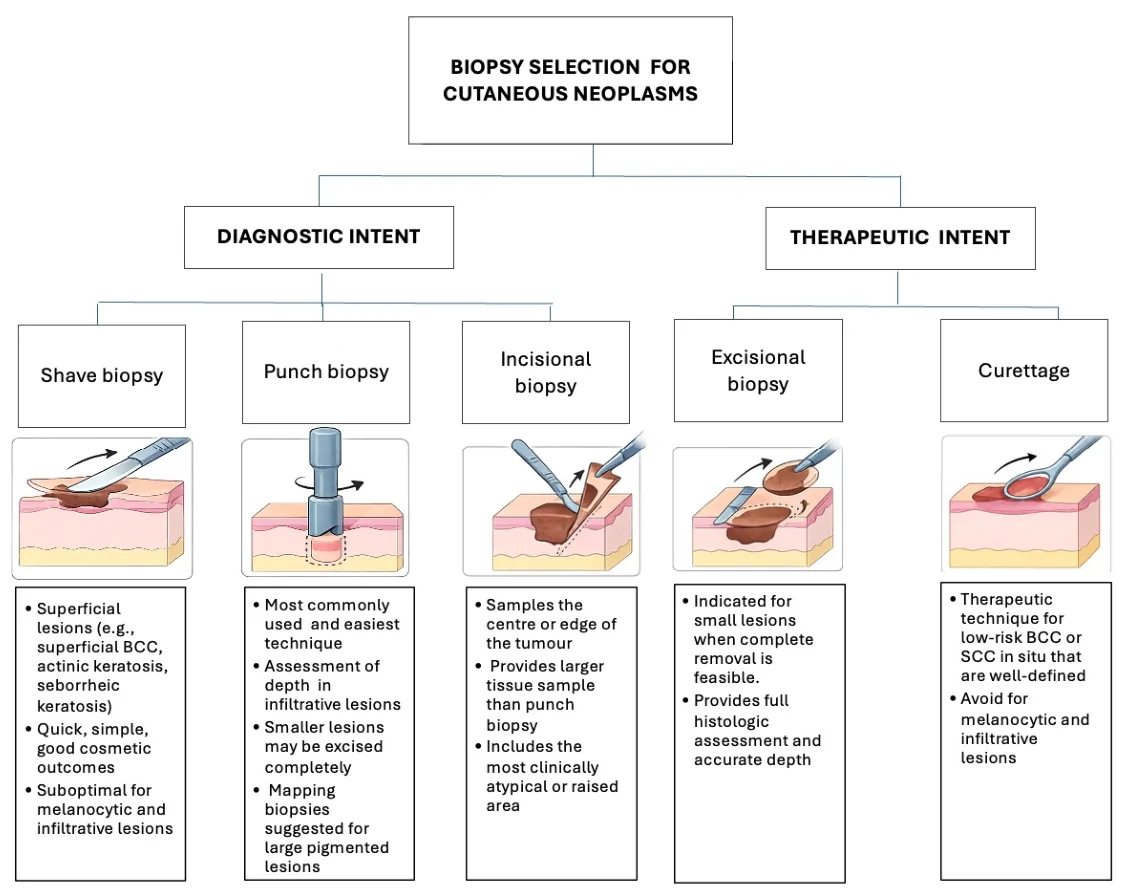
Biopsy selection algorithm for cutaneous neoplasms (created with FIGURELABS.AI).

The “Biopsy First, Plan Surgery Second” goal is to obtain a representative tissue sample for accurate histopathologic diagnosis and subsequent treatment planning. The chosen biopsy method is determined by the type and site of the skin lesion, the suspected diagnosis, the required histological parameters, and the patient preferences (Figure 1 and Figure 2) [4].

### 2.1. Procedures with Diagnostic Intent

#### 2.1.1. Shave Biopsy

Shave biopsy is a superficial sampling technique primarily used for lesions with a predominantly epidermal component (e.g., superficial basal cell carcinoma, actinic keratosis, or seborrheic keratosis). The procedure is quick, technically simple, and generally associated with good cosmetic outcomes and minimal morbidity [4,5].

The main disadvantage of shave biopsies is the risk for limited histological assessment. This is particularly problematic in malignant melanoma, where crucial parameters such as Breslow thickness, ulceration, mitotic rate, and perineural invasion may not be fully evaluated. For this reason, superficial shave biopsies should generally be avoided when melanocytic lesions or other deeply invasive tumours are clinically suspected.

#### 2.1.2. Punch Biopsy

Punch biopsies are the most commonly used and easiest technique to perform, with the least morbidity. While ideally it is desirable for a tumour or a melanocytic lesion to be sampled in its entirety, for larger lesions, this may not be feasible and a punch biopsy may be obtained from the thickest/most nodular part. Nonetheless, lesions smaller than the punch diameter may be excised completely. For large pigmented lesions, mapping biopsies may also be considered, bearing in mind that the specimen may not be entirely representative, with a risk of missing the area of deepest invasion or of focal malignancy.

#### 2.1.3. Incisional Biopsy

This involves obtaining tissue from the centre or edge of the tumour, and can be used to sample a significant part of large lesions. Similar to punch biopsies, incisional biopsies should include the most raised or clinically atypical area of a large lesion.

### 2.2. Procedures with Therapeutic Intent

#### 2.2.1. Curettage

Curettage is primarily a therapeutic technique used for low-risk keratinocytic neoplasms, particularly superficial or nodular basal cell carcinoma (BCC) and squamous cell carcinoma in situ. The technique relies on the friability of tumour tissue relative to surrounding dermis and is most suitable for well-defined tumours.

**Figure 3:**
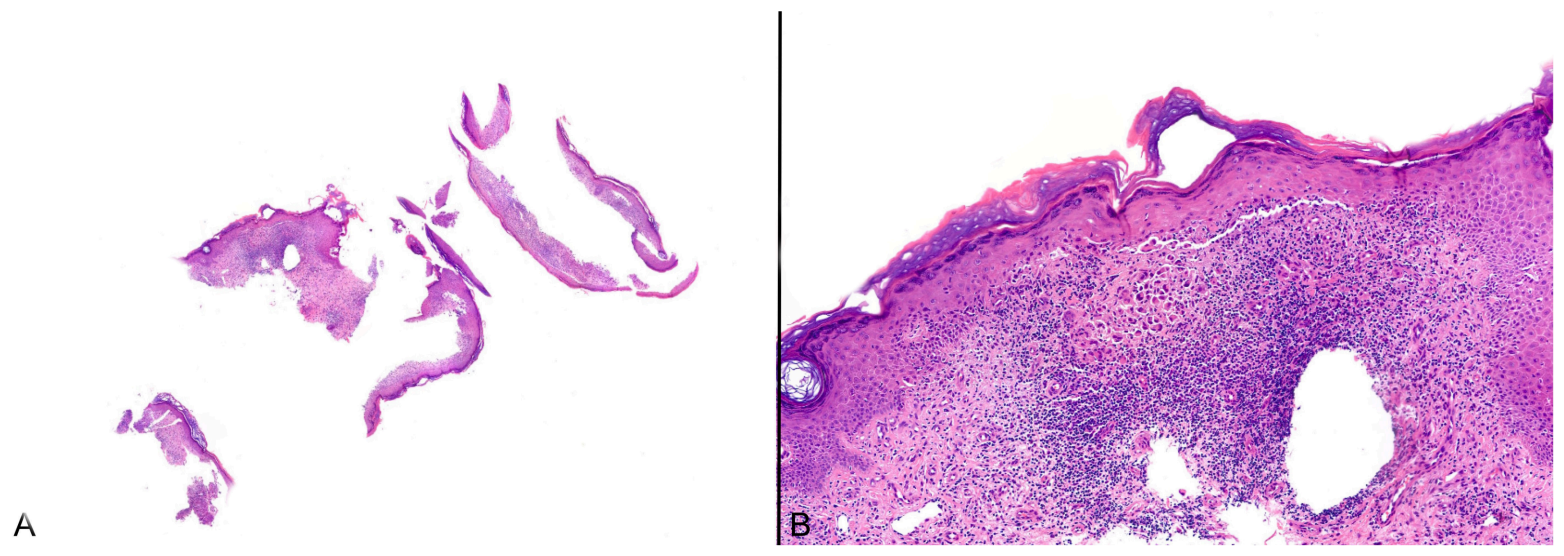
(**A**,**B**) An unintended curettage of a malignant melanoma, where the clinical impression had been felt to be Bowen’s disease. The fragmented specimen limits assessment of architecture and Breslow thickness. This figure is simple original microscopic photos taken directly from regular cases. Not previously published or illustrates new conclusion.

The quality of the specimen can be operator-dependent. The often suboptimal orientation tends to hamper the assessment of important histopathological parameters such as invasion, tumour thickness and margins. It is generally advisable to avoid curettage for high-risk histological subtypes and infiltrative lesions due to the risk of recurrence. Curettage should also be avoided for melanocytic lesions; however, this may occur unintentionally when a non-suspected flesh-coloured lesion is curetted (Figure 3).

#### 2.2.2. Excisional Biopsy (Excision in Toto)

Technique in which the biopsy is intended as curative treatment, with full-thickness tumour removal with narrow (2–3 mm) margins of clinically normal skin. Excisional biopsies should be considered as a first option when malignancy is strongly suspected and is the technique of choice in suspected melanoma, especially for smaller lesions.

Excisional biopsies have the advantage of providing the entire lesion from the outset for full microscopic assessment, including the architecture, microstaging (depth, ulceration) and diagnosis.

## 3. Definitive Surgical Treatment and Margin Assessment

The goal of definitive excision is the complete removal of the tumour with histologically negative margins to minimise recurrence. If the lesion has been previously sampled, preoperative planning based on the biopsy report is imperative.

**Figure 4:**
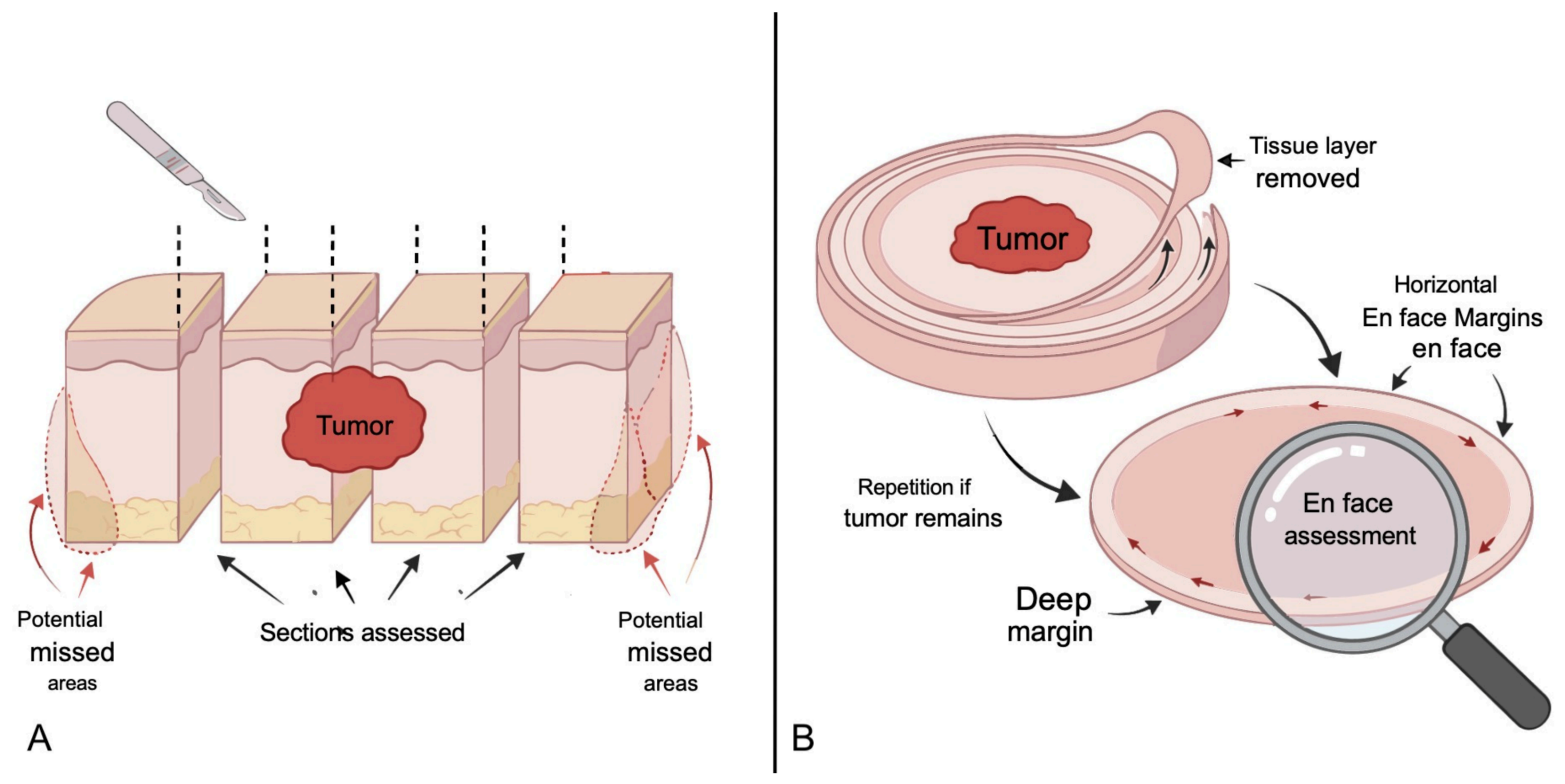
Comparison of skin excision grossing methods: (**A**) bread-loaf sectioning vs. (**B**) Mohs surgery with en-face margin assessment (created with FIGURELABS.AI).

Standard excisions are processed with bread-loaf histological sectioning, which only examines a fraction of the true margin, so “negative margins” are probabilistic rather than absolute. In contrast, Mohs Micrographic Surgery (MMS) allows complete examination of 100% of the surgical margin, through peripheral and deep en-face margin assessment (Figure 4). MMS is the gold standard for high-risk or cosmetically sensitive tumours [6,7,8].

## 4. Margin Guidelines for Melanocytic Lesions

**Figure 5:**
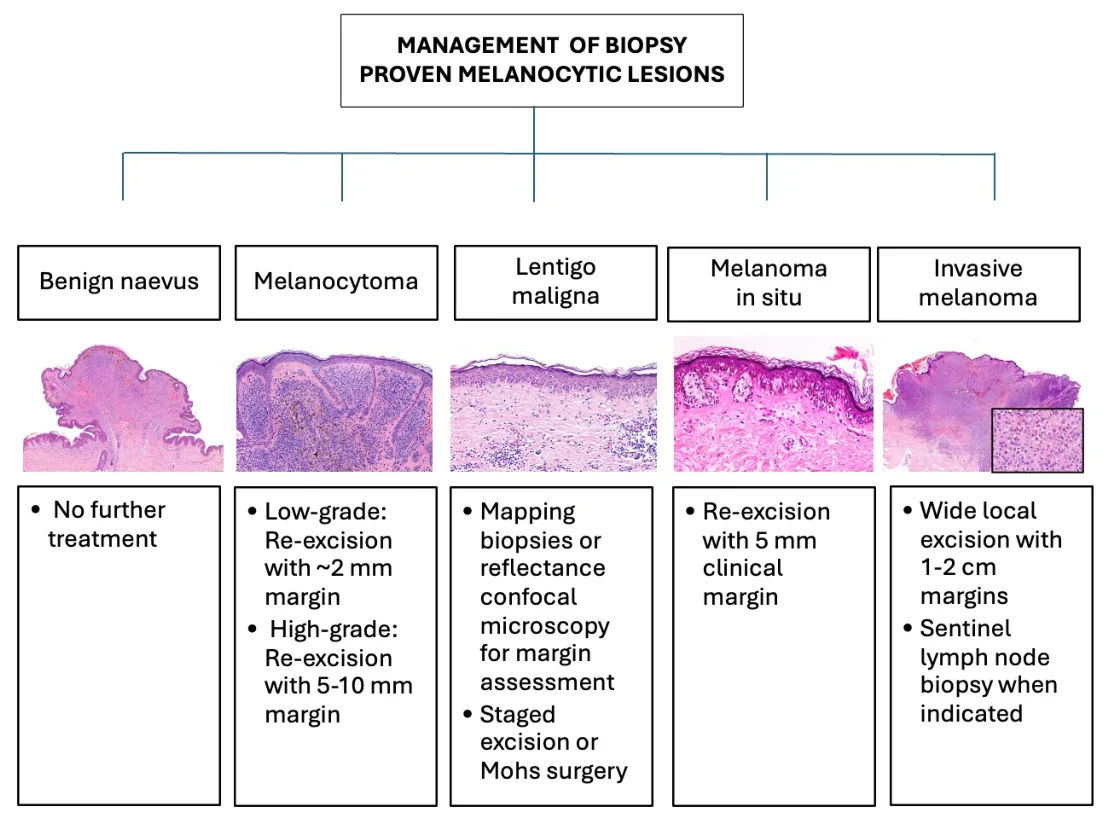
Management algorithm for melanocytic lesions. This figure is simple original microscopic photos taken directly from regular cases. Not previously published or illustrates new conclusion.

A narrow diagnostic excisional biopsy from the outset for melanocytic lesions is the method of choice when feasible. For larger lesions, either an incisional biopsy or a punch biopsy can be done to include the most clinically concerning area. Mapping punch biopsies can be performed to delineate the extent of poorly defined lesions. The specimen should extend into subcutaneous fat to ensure accurate depth measurement. Superficial shave biopsies should be avoided. Surgical margins guidelines are summarised in Figure 5.

Therapeutic excision margins for melanoma are dependent upon stage but may vary according to national guidance (NCCN/EADO/BAD/NICE). In the UK, the National Institute for Health and Excellence (NICE) guidelines recommend a clinical margin of at least 5 mm at initial excision for in situ melanoma. If the lesion extends to the margins, further management should be discussed in a multidisciplinary setting.

Histopathologically proven invasive melanoma should be further excised with a 1–2 cm margin. This margin should be around the biopsy scar, taking into account the original margins [9,10,11]. In NICE guidelines, sentinel lymph node procedure should be considered in pT1b stage if there are high-risk features (ulceration, two or more mitoses, or lymphovascular invasion) and stage pT2a or above.

### 4.1. Lentigo Maligna and Lentigo Maligna Melanoma

Managing lentigo maligna (LM) and lentigo maligna melanoma (LMM) can be quite challenging due to frequent subclinical extension. Pre-surgical mapping punch biopsies may help outline the extent of the disease. Reflectance confocal microscopy (RCM), an imaging technique that allows assessment of cellular detail of the skin in vivo, is also useful in pre-surgical estimation of LM surgical margins [12]. RCM is currently approved for use by both the FDA and EMA for the USA and Europe, respectively [13,14].

Treatment choice for LM would depend on available resources as well as the patient preference. Surgical options include staged excision, MMS, or “slow Mohs.” The latter, unlike standard, same-day MMS, uses paraffin-embedded sections rather than frozen sections, allowing for the use of immunohistochemical markers if needed.

Although not as established as MMS, some alternative techniques have been described and can potentially be considered for lentiginous proliferations, particularly when MMS is not readily available. One such example is the so-called “spaghetti technique”. This involves removing a thin strip of skin that outlines the lesion. The defect is sutured and the removed tissue is examined on histopathology. If the pathology is positive, the strip removal is repeated only for the involved segments.

Once the histological margins are confirmed clear, the primary tumour is resected with a minimal margin, and reconstruction is performed [15].

As LM is a poorly demarcated lesion, PRAME has emerged as a useful tool when assessing excision margins. However, scattered PRAME-positive cells can be seen in chronically sun-damaged skin, and when not associated with cytologic atypia, careful clinicopathologic correlation would be paramount [16].

### 4.2. Intermediate Melanocytic Lesions

The current WHO classification defines melanocytomas as genetically intermediate melanocytic lesions, such as WNT-activated melanocytoma, BAP1-inactivated melanocytoma and pigmented epithelioid melanocytoma (PEM), while restricting the term naevus for lesions solely harbouring the initiating molecular aberration [17].

The current MPATH-Dx version 2.0 schema essentially classifies all melanocytomas as class II lesions, with low risk of progression to invasive melanoma, with recommendation to re-excision with margins < 1 cm^2^, when margins are positive in adequately sampled lesions [18].

To improve the understanding on management approach to the melanocytoma category for clinicians, the ESP, EORTC, and EURACAN consensus recommends a 2 mm margin for low-grade melanocytomas and a 5–10 mm margin for high-grade melanocytomas, based on atypical cytological and architectural features. PEMs, in this classification, are considered high-grade lesions by definition, with a recommended 5–10 mm margin [19].

## 5. Margin Guidelines for Non-Melanoma Skin Cancers

### 5.1. Basal Cell Carcinoma

The decision on BCC excision margins requires assessing if the risk of lesion recurrence is low vs. high risk. This includes both clinical and histopathological criteria. Clinically, high-risk BCCs may be ill-defined or a recurrent lesion, of a size ≥ 20 mm on the trunk and extremities, or of any size on other sites, if they occur at a previous site of radiotherapy, or have a patient history of immunosuppression [20,21,22]. Histologically high-risk BCCs are either of aggressive growth patterns, have perineural invasion or involve/are close to <1 mm of the peripheral margin.

Table 1 summarises management according to low-risk and high-risk BCC. For low-risk BCC, surgical treatment options include C&C and excision with a 2–4 mm clinical margin depending on institutional and national guidelines. High-risk BCC are treated with wider margin excisions (5–10 mm) or treated in sensitive anatomical sites (such as head and neck) with MMS [20].

Optical coherence tomography (OCT) and dermoscopy-guided high-frequency ultrasound are recently described imaging modalities for margin assessment for BCC. However, their implementation is currently limited as they are not widely available [23,24].

### 5.2. Squamous Cell Carcinoma (SCC)

Guidelines include an additional very-high-risk category in SCC, with higher risk for local recurrence or metastasis. Clinical criteria are mostly similar to those of BCC, with the addition of the presence or absence of neurological symptoms or rapid tumour growth and >40 mm size. Histological criteria are based on differentiation and subtype, depth and level of invasion, and lymphovascular or perineural invasion (Table 2).

Similar to BCC, treatment options for low-risk SCC include C&C, shave, or excision with a 4–6 mm clinical margin. High-risk SCC requires discussion at multidisciplinary meetings. Surgical options include a wider excision (5–10 mm clinical margin) or consideration of MMS [25].

## 6. Conclusions

In summary, a nuanced, pathology-informed approach optimises skin tumours treatment and cosmetic outcomes. The initial biopsy choice can be based on lesion size, location, and clinical suspicion. The subsequent definitive surgery can be planned according to the provided histopathologic data, with the appropriate margin guidelines applied. Specialised techniques such as MMS or staged excisions can be considered for high-risk or complex cases.

## Figures and Tables

**Table 1:** Management of BCC according to risk stratification.

Parameters	Low Risk	High Risk
**Clinical**		
Location/size	Trunk/extremities < 20 mm	Trunk/extremities ≥ 20 mm, head and neck, hands, feet, pretibial and anogenital areas (any size)
Borders	Well defined	Poorly defined
Primary vs. recurrent	Primary	Recurrent
Immunosuppression	No	Yes
Site of prior radiotherapy	No	Yes
**Pathological**		
Subtype	Superficial or nodular	Aggressive pattern (infiltrative, morpheaform, micronodular, basosquamous, carcinomsarcomatous)
Perineural involvement	No	Yes
**Margin guidelines**	4 mm	5–10 mm MMS

Low vs. high-risk basal cell carcinoma (adapted from [20,21,22]).

**Table 2:** Management of SCC according to risk stratification.

Parameters	Low Risk	High Risk	Very High Risk
**Clinical**			
Location/size	Trunk/extremities < 20 mm	Trunk/extremities ≥ 20 mm, head and neck, hands, feet, pretibial and anogenital areas (any size)	40 mm (any site)
Borders	Well defined	Poorly defined	
Primary vs. recurrent	Primary	Recurrent	
Immunosuppression	No	Yes	
Site of prior radiotherapy	No	Yes	
Rapid tumour growth	No	Yes	
Neurological symptoms	No	Yes	
**Pathological**			
Differentiation	Well or moderate		Poor
Subtype	-	Adenosquamous or sarcomaotoid in any part of the tumour	Adenosquamous or sarcomaotoid in any part of the tumour
Depth, thickness or level of invasion	<2 mm thickness and no invasion beyond subcutis	2–6 mm depth and no invasion beyond subcutis	>6 mm or invasion beyond subcutis
Perineural involvement	No	Yes	Yes (deep to the dermis or >0.1 mm in size)
Lymphovascular involvement	No	No	Yes
Histological margins	≥1 mm	0 or close < 1 mm	0 or close < 1 mm
Margin guidelines	4–6 mm	MDT discussion 5–10 mm MMS	MDT discussion 5–10 mm MMS

Squamous cell carcinoma stratification (adapted from [25]).

## Data Availability

No new data were created or analysed in this study. Data sharing is not applicable to this article.
